# RNA modifications and their role in gene expression

**DOI:** 10.3389/fmolb.2025.1537861

**Published:** 2025-04-25

**Authors:** I. Made Artika, Rini Arianti, Máté Á. Demény, Endre Kristóf

**Affiliations:** ^1^ Department of Biochemistry, Faculty of Mathematics and Natural Sciences, Bogor Agricultural University, Bogor, Indonesia; ^2^ Laboratory of Cell Biochemistry, Department of Biochemistry and Molecular Biology, Faculty of Medicine, University of Debrecen, Debrecen, Hungary; ^3^ Universitas Muhammadiyah Bangka Belitung, Pangkalpinang, Indonesia; ^4^ Department of Medical Chemistry, Faculty of Medicine, University of Debrecen, Debrecen, Hungary

**Keywords:** RNA, RNA modification, epitranscriptomics, gene expression, RNA editing, therapeutic development

## Abstract

Post-transcriptional RNA modifications have recently emerged as critical regulators of gene expression programs. Understanding normal tissue development and disease susceptibility requires knowledge of the various cellular mechanisms which control gene expression in multicellular organisms. Research into how different RNA modifications such as in N6-methyladenosine (m^6^A), inosine (I), 5-methylcytosine (m^5^C), pseudouridine (Ψ), 5-hydroxymethylcytosine (hm^5^C), N1-methyladenosine (m^1^A), N6,2′-O-dimethyladenosine (m^6^Am), 2′-O-methylation (Nm), N7-methylguanosine (m^7^G) etc. affect the expression of genes could be valuable. This review highlights the current understanding of RNA modification, methods used to study RNA modification, types of RNA modification, and molecular mechanisms underlying RNA modification. The role of RNA modification in modulating gene expression in both physiological and diseased states is discussed. The potential applications of RNA modification in therapeutic development are elucidated.

## 1 Introduction

Gene expression ─ a cellular process by which the information encoded in a gene is converted into a functional gene product ─ is tightly controlled at multiple layers to ensure production of appropriate level of each gene product, such as a protein (Lackner et al., 2007). The vast majority (up to 90%) of eukaryotic genomes is pervasively transcribed (Kaikkonen et al., 2011). It is interesting to note, however, that only about 1.5% of the human genome represents protein-coding genes which are transcribed into messenger RNA (mRNA), while the rest, about 98.5%, consists of non-protein-coding DNA sequences, which are transcribed into non-coding RNA (ncRNA) molecules. Compared to that of mRNAs, the transcription levels of most of ncRNAs are significantly lower indicating that ncRNAs primarily serve regulatory functions (Kaikkonen et al., 2011; Richard Boland, 2017). The ncRNAs can be further classified into infrastructural ncRNAs and regulatory ncRNAs. The infrastructural ncRNAs include ribosomal RNA (rRNA), transfer RNA (tRNA), small nuclear RNA (snRNA), and small nucleolar RNA (snoRNA). The regulatory ncRNAs consist of microRNAs (miRNAs), Piwi-interacting RNAs (piRNAs), small interfering RNAs (siRNAs), and long non-coding RNAs (lncRNAs) (Kaikkonen et al., 2011). Circular RNA (circRNA) is a novel type of ncRNA ubiquitously expressed in eukaryotic cells during posttranscriptional processes. This type of RNA forms covalent-closed continuous loops without 5′ to 3′ polarities and poly (A) tails. With the aid of high-throughput sequencing methods, numerous circRNAs have been discovered in humans, animals, and plants (Wang et al., 2017). Other classes of RNA molecule, promoter-associated RNAs (PARs) and enhancer RNAs (eRNAs), have been identified through high-throughput sequencing of RNA molecules (Kaikkonen et al., 2011).

Both coding and ncRNA can undergo biochemical modification co- or post-transcriptionally which diversify RNA molecules and affect their cellular function. Apart from the well-known 5′ capping and 3′ polyadenylation, numerous internal nucleoside modifications also occur in RNA transcripts which exhibit a profound impact on their biochemical characteristics (Roundtree et al., 2017; McCown et al., 2020). When post-transcriptional RNA modification changes the nucleotide sequence in the coding region of a primary transcript which may change the amino acid sequence of the encoded protein, the alteration is classed as RNA editing. Thus, RNA editing is part of RNA modification and can be defined as posttranscriptional alterations of RNA molecules through insertion, deletion, or modification of nucleotides (except RNA processing events such as splicing, capping, or polyadenylation) which bring about differences between the actual genomic sequence and the corresponding RNA sequence (Xu and Zhang, 2014). RNA editing includes base modifications such as deamination of adenosine (A) to inosine (I) and deamination of cytidine (C) to uridine (U). These base alterations are catalyzed by deaminases which act as editors. The A-to-I conversion is the most prevalent type of RNA editing in animal cells. In humans, more than 4.6 million A-to-I modification sites have been identified (Christofi and Zaravinos, 2019; Lo Giudice et al., 2020). The majority of RNA editing sites are located in non-coding regions and only a small proportion occurs in the coding sequences of RNA, thus altering the amino acid sequence and the function of their encoded proteins (Zinshteyn and Nishikura, 2009). RNA editing has been linked to various human diseases such as autoimmune and inflammatory pathologies, neurodegenerative and psychiatric disorders, and cancer (Lo Giudice et al., 2020).

**FIGURE 1:**
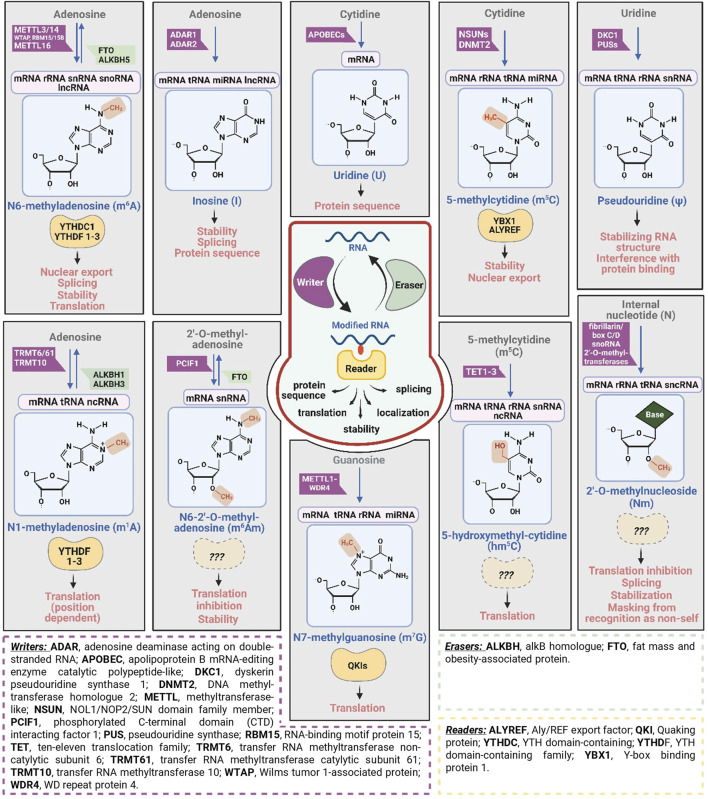
The RNA modifications discussed in this article. The main writer (purple boxes) and eraser (green boxes) enzymes and the known reader proteins (yellow shapes) are shown together with the modifications’ recognized effect and their distribution in different RNA types. Created in BioRender. Demeny (2025) https://BioRender.com/n91u871.

RNA modifications have been found to take place in all living cells (Zhao et al., 2017) as well as in both DNA and RNA viruses (Baquero-Perez et al., 2021). Notably, tRNAs have been found to be the most heavily modified, each of which has on average 13 modifications. Similarly, rRNAs are also frequently modified although, to a lesser extent than tRNAs (Arzumanian et al., 2022). Chemical modifications found in human rRNA include 2′-O-methylation, pseudouridines (Ψs), and base methylations. The biogenesis of rRNA is prevented in the absence of internal Ψs and 2′-O-methylated sugars, indicating the crucial roles of rRNA chemical modifications (Roundtree et al., 2017). Currently, more than 170 different types of posttranscriptional RNA modification have been identified (Wiener and Schwartz, 2021; Cappannini et al., 2024; Xuan et al., 2024). Of these, the N6-methyladenosine (m^6^A), I, 5-methylcytosine (m^5^C), Ψ, 5-hydroxymethylcytosine (hm^5^C), N1-methyladenosine (m^1^A), N6,2′-O-dimethyladenosine (m^6^Am), 2′-O-methylation (Nm), and N7-methylguanosine (m^7^G) (Figure 1) are among the most common RNA modifications (Song and Yi, 2017; Roundtree et al., 2017; Cai et al., 2023). The m^6^A RNA methylation is the most prevalent RNA modification (He and He, 2021). The collection of RNA modifications presents in a living organism or a virus is termed epitranscriptome, and the field that studies RNA modifications is referred to as epitranscriptomics (Song and Yi, 2017; Xiong et al., 2017). This newly emerged field is progressing rapidly along with the advancement of both experimental and computational methods for deciphering RNA modifications (Primac et al., 2022). Mutations in genes encoding enzymes for RNA modifications have been linked to different types of human diseases (Jonkhout et al., 2017).

Several databases related to RNA modifications have been developed (Table 1). These include MODOMICs (Cappannini et al., 2024), RMBase (Xuan et al., 2024), RMDisease V2.0 (Song B. et al., 2023), and RNAMDB (Cantara et al., 2011). MODOMICS is a comprehensive database on the chemical structures of modified RNA nucleosides, their biosynthetic pathways, the position of modified residues in RNA sequences, and enzymes responsible for RNA modifications (Cappannini et al., 2024). RMBase provides various resources and tools useful for studying RNA modifications. This database enables integrated analysis of diverse RNA modification profiles and makes possible exploration of transcriptome-wide landscape, biogenesis, molecular interactions, and functions of RNA modifications (Xuan et al., 2024). RMDisease V2.0 is an updated database of genetic variants which affect RNA modifications with disease implications. This database is intended to unmask the link between disease-related genetic variants and their epitranscriptome alterations (Song B. et al., 2023). The RNAMDB database provides information on different aspects of naturally occurring RNA modifications such as chemical structure, common name and symbol, elemental composition, and mass (Cantara et al., 2011). In addition, a comprehensive database of RNA modifying enzymes has also been developed. This database, called RNAME, lists more than 21,000 RNA modification enzymes from 456 species and is aimed to facilitate studies on RNA modifications (Nie et al., 2022). A knowledgebase for m^6^A epitranscriptome, m^6^A-Atlas v2.0, has also been created (Liang et al., 2024). Considering the critical roles of RNA modification throughout development, and the current intense research on RNA modification, this review highlights the recent studies and progress related to dynamics of RNA modification. Current knowledge of RNA modification and their important roles on regulation of gene expression in both physiological and diseased states are addressed.

**TABLE 1:** Databases related to RNA modifications.

Database	Type of RNA modification	Database Function(s)	References	Links
MODOMICs	Many types of RNA modification	A catalog of modified residues, enzymes responsible for reaction, RNA modification pathway, sequence of modified RNA, links to diseases and relevant publications	Cappannini et al. (2024)	https://iimcb.genesilico.pl/modomics/
RMBase	Many types of RNA modification	Integrated analysis of diverse RNA modification profiles. Facilitates transcriptome exploration (landscape, biogenesis, interactome, and function)	Xuan et al. (2024)	http://bioinformaticsscience.cn/rmbase/
RMDisease V2.0	m^6^A, m^5^C, m^1^A, m^5^U, Pseudouridine (Ψ), m^6^Am, m^7^G, A-to-I, ac^4^C, Am, Cm, Um, Gm, hm^5^C, D, and f^5^C	As a database of genetic variants that affect RNA modifications with disease and trait implication	Song B. et al. (2023)	http://www.rnamd.org/rmdisease2
RNAMDB	Many types of naturally occurring RNA modifications	Provides RNA modification related-information (structure, common name and symbol, elemental composition and mass, CA registry numbers and index name, phylogenetic source, type of RNA species, and references)	Cantara et al. (2011)	http://rna-mdb.cas.albany.edu/RNAmods/
RNAME	Cap, I, m^1^A, m^6^A, m^5^C, Ψ, and m^7^G	A collection of RNA modification enzymes (more than 21,931 manually curated writers, readers and erasers) from 456 species covering animals, plant, and fungi	Nie et al. (2022)	https://chenweilab.cn/rname/
m^6^A-Atlas v2.0	m^6^A	Resources for the m^6^A epitranscriptome among multiple species	Liang et al. (2024)	http://rnamd.org/m6a

## 2 Methods to study RNA modification

Improved methodologies have stimulated research and led to better understanding of RNA modification. Different techniques with diverse strategies have been employed to detect, map, quantify, analyze, and illuminate cellular function of RNA modifications (McMahon, 2021). These include microarray (Hiley et al., 2005), restriction fragment length polymorphism (RFLP) (Wulff et al., 2017), mass spectrometry (You and Yuan, 2021; Zhang et al., 2021), nuclear magnetic resonance (NMR) (Gato et al., 2021), polymerase chain reaction (PCR) (Elliott and Holley, 2021; Olazagoitia-Garmendia and Castellanos-Rubio, 2021), Northern blot (Cirzi and Tuorto, 2021), enzymatic (Czekay et al., 2021), next-generation sequencing (NGS) (Marchand et al., 2021; Cullen and Tsai, 2021), nanopore direct RNA sequencing (Liu H. et al., 2021; Jain et al., 2021), and CRISPR-Cas9 (Liu and Qian, 2021) methods. In addition, bioinformatics tools have been applied to analyze RNA modification data (Manfredonia and Incarnato, 2021; Liu Q. et al., 2021).

The microarray technique can be used to differentiate RNA molecules, both with and without modification based on the binding of the RNA molecules to the probes on the array. Using this method, Hiley and coworkers could detect at least five different RNA modifications (Hiley et al., 2005). A protocol based on differential enzymatic digestions coupled with liquid chromatography-tandem mass spectrometry (LC-ESI-MS/MS) has been developed and applied to identify internal m^7^G in mRNA of different types of human cell. This protocol is also applicable for detection and quantification of m^7^G at the 5′ cap of mRNA (You and Yuan, 2021). A method based on general LC-MS has also been developed for direct and *de novo* sequencing of purified RNAs, containing both canonical and modified nucleotides such as Ψ and m^5^C (Zhang et al., 2021). Furthermore, in order to study tRNA maturation, a method using NMR has been developed to directly monitor the introduction of biochemical modifications in the process of tRNA maturation (Gato et al., 2021).

Detecting 2′-O-methylation (Nm) on specific mRNA transcripts is technically challenging because mRNAs are much less abundant compared with rRNA. A strategy based on quantitative PCR in conjunction with reverse transcription at a low level of dNTPs has been developed and was demonstrated to be sensitive to detect changes to Nm modification of mRNA (Elliott and Holley, 2021). Similarly, site-specific detection and quantification of m^6^A is technically difficult. A simple reverse transcription-qPCR-based assay has been developed which can be implemented for the relative quantification of candidate m^6^A regions. This strategy takes advantage of the reduced capacity of BstI enzyme to retrotranscribe m^6^A residues (Olazagoitia-Garmendia and Castellanos-Rubio, 2021). A reverse transcription polymerase chain reaction (RT-PCR) and gel electrophoresis-based method has been developed to detect and quantify Ψ RNA modification. This simple technique was found to be helpful in validating Ψ sites identified by high throughput sequencing, quantifying Ψ levels in mRNA and lncRNA, and effectively elucidate the mechanisms and function of the Ψ modification (Zhang and Pan, 2022).

A protocol using N-acryloyl-3-aminophenylboronic acid (APB) during Northern blot has been developed for fast and reliable detection of queuosine (Q) tRNA modification. This assay allows separation of Q-modified tRNA from unmodified tRNA and quantification can be carried out using Northern blot analysis (Cirzi and Tuorto, 2021). The Northern blot technique has also been applied for detection of RNA modifications by using antibodies against modified nucleosides. The development of this immuno-Northern blotting approach was intended to facilitate studies on RNA modifications and metabolism (Mishima et al., 2015).

Besides the classical approaches, NGS-based methods have been applied to study RNA modifications. A method called AlkAniline-Seq was developed and found to be fast and efficient for simultaneously mapping two different RNA modifications, the m^7^G and m^3^C (Marchand et al., 2021). The high-throughput NGS can also be employed to identify antibody-bound modified transcripts. Based on this principle, a method termed “photoactivatable ribonucleoside-enhanced cross-linking and immunoprecipitation” (PAR-CLIP) has been developed for mapping various RNA modifications for which specific antibodies against the RNA modifications are available (Cullen and Tsai, 2021). The advent of NGS technologies has accelerated research on RNA editing. Recently, a computational method for profiling the editome (the entire RNA editing in a genome) from single-cell RNA sequencing (scRNA-seq) data has been developed. This tool is useful for detecting RNA editing events in functionally heterogeneous cell populations (Wu et al., 2023a).

Due to its abundance and critical cellular roles of m^6^A RNA modification, a method which enables identification of m^6^A sites in the whole transcriptome of single cells is required to study m^6^A contribution to normal cellular function and disease pathogenesis. A method called deamination adjacent to RNA modification targets sequencing (DART-seq) was found to be applicable for transcriptome-wide profiling of m^6^A sites to reveal different m^6^A signatures and mRNA methylation heterogeneity in single cells (Tegowski et al., 2022). In addition, to improve resolution and allow quantitative detection of m^6^A, a method named “evolved TadA-assisted N6-methyladenosine sequencing” (eTAM-seq) has been created. It is an enzyme-assisted sequencing platform which detects and quantifies m^6^A by global adenosine deamination (Xiao et al., 2023).

The high-throughput sequencing techniques either based on antibodies, enzymes, or novel chemistry have been employed to study m^6^A and Ψ RNA modification (Zhang et al., 2023a). Recently, a chemical assisted-method called “glyoxal and nitrite-mediated deamination of unmethylated adenosines” (GLORI) has been developed and used for absolute quantification of single-base m^6^A methylation in the mammalian transcriptome (Liu C. et al., 2023). Similarly, a sensitive and convenient chemical assisted-method termed PRAISE was developed to measure transcriptome-wide Ψ (Zhang et al., 2023b). Methods for the precise mapping of individual RNA modifications throughout the transcriptome are critical in studying roles of a specific transcriptome. A method called hydrazine-aniline cleavage sequencing (HAC-seq) has been developed and applied to specifically map m^3^C throughout a transcriptome. This novel method can be used to reveal the m^3^C methylome in various cells and tissues (Cui et al., 2021). Recently, a specific and sensitive technique called “m^6^Am-seq” has been introduced to investigate the prevalence, topology, and dynamics of m^6^Am in the human transcriptome. This technique is based on a selective demethylation reaction to achieve specific and sensitive detection of m^6^Am (Sun et al., 2021).

The advancement of the nanopore direct RNA sequencing (dRNA-seq) technique has further improved the methodology for identification of posttranscriptional RNA modification. This technique enables direct sequencing of full-length native RNA molecules without the need of a reverse-transcription or amplification step and can provide a comprehensive picture of individual RNA molecules as their existence in cells. More importantly, this emerging method allows detection of different nucleotide modifications present in the native RNA molecules on single-read level data using a portable device (Leger et al., 2021; Zhao et al., 2022; Jain et al., 2022). An algorithm, called Epi Nano has been developed to detect RNA base modifications, such as m^6^A, from data generated using nanopore dRNA-seq (Liu H. et al., 2021). A protocol for sequencing canonical and modified nucleotides of human rRNA using nanopore dRNA-seq has been established (Jain et al., 2021). Recently, a study comparing the use of dRNA-seq and methylated RNA immunoprecipitation and sequencing (MeRIP-seq) in detecting m^6^A modification in ncRNAs of glioblastoma suggested that MeRIP-seq is preferable for a preliminary m^6^A screening study, as it exhibits a higher lncRNA coverage, while the dRNA-seq is more useful for in depth analysis of m^6^A quantity and exact location. Of note, MeRIP-seq is the most common method for m^6^A detection (Krusnauskas et al., 2023).

Analysis of data generated from high-throughput sequencing techniques has been the main bottleneck in experiments using these assays. Systematic identification of different types of RNA-modification sites remains a major challenge. More than 20 computational methods have been developed to map RNA-modification sites (Chen et al., 2020). A generalized toolkit for the analysis of NGS-based RNA posttranscriptional modification mapping experiments has been generated (Manfredonia and Incarnato, 2021). A protocol for identification and annotation of individual RNA modifications throughout the transcriptome has also been created to promote research on the roles of the epitranscriptome in the control of gene expression and other cellular processes (Liu Q. et al., 2021). The availability of large datasets of transcriptomics has led to the increase of application of machine learning approaches to identify RNA modifications (El Allali et al., 2021). Considering the pivotal roles of m^6^Am RNA modification, a Catboost-based model, using machine learning algorithms was developed for predicting the m^6^Am sites on mRNA (Liu Z. et al., 2023). Machine learning has also been used to predict genes linked to RNA methylation pathways (Tsagkogeorga et al., 2022). An effective computational method, iRNA5hmC, which is complementary to the high-throughput sequencing technologies, has been introduced for identification of RNA hm^5^C sites using machine learning (Liu Y. et al., 2020). A predictor named iRNA5hmC-HOC based on a high-order correlation information method has been proposed for identification of hm^5^C sites (Zou, 2022).

## 3 Types of RNA modification

RNA modifications are dynamic processes, catalyzed by a series of specific modifying enzymes or proteins, which are based on their functions can be grouped into the so-called writer, eraser, and reader categories (Figure 1). Writers are enzymes which play roles in installing chemical modifications into RNA molecules, while those functions in removing the chemical modifications are termed erasers. Proteins which recognize the chemical marks are called readers. These play a critical role in transducing signal for downstream functions (Ontiveros et al., 2019; Nie et al., 2022; Qiu et al., 2023). Currently, our detailed understanding of RNA modifying enzymes and their mechanisms of action is limited because only a small number of experimentally validated RNA modification enzymes are documented (Nie et al., 2022). The majority of internal RNA modifications occur post-transcriptionally. Notably, co-transcriptional modifications have been documented for m^6^A and Ψ (Gilbert and Nachtergaele, 2023). The types of chemical modification that decorate RNA molecules are diverse which include methylation, deamination, isomerization, thiolation, glycosylation, transglycosylation, attachment of amino acid, addition of sugar, *etc.* (Jackman and Alfonzo, 2013; Ontiveros et al., 2019). These modifications may affect folding, Watson-Crick base pairing, 3D structure, molecular flexibility, molecular interaction with other molecules, molecular stability, and biological function of the modified RNAs (Ontiveros et al., 2019; Adamopoulos et al., 2023).

### 3.1 N6-methyladenosine (m^6^A)

So far, the molecular mechanism underlying the m^6^A RNA modification system is the most well studied and hence well understood among hundreds known types of RNA modification (Nie et al., 2022). The m^6^A modifications are found on mRNA, tRNA, rRNA, snRNA, and ncRNAs such as lncRNAs, miRNAs, and circRNAs (Zhou et al., 2020; Bedi et al., 2023). It is enriched near stop codons and 3′-untranslated terminal regions (UTRs) (Zhou et al., 2020). The m^6^A modification results from a methylation reaction at the N6-position of adenosine in the RNA molecule catalyzed by a complex writer-protein comprised of methyltransferase-like (METTL) 3, METTL5, METTL14, METTL16 and their cofactors such as Wilms tumor 1-associated protein (WTAP), RNA-binding motif protein 15 (RBM15/15B), Cbl proto-oncogene-like 1 (CBLL1; also named HAKAI), zinc finger CCCH-type containing 13 (ZC3H13), and Vir-like m^6^A methyltransferase-associated (VIRMA; also termed KIAA1429) (Zhou et al., 2020). METTL3 and METTL14 form a heterodimeric complex forming the core methyltransferase that catalyzes the m^6^A modification. METTL3 is the catalytic subunit of the complex responsible for binding the co-substrate S-adenosyl methionine (SAM), while METTL14 functions as structural support for METTL3 and is involved in mRNA binding (Zhou et al., 2020; Jiang et al., 2021; Bedi et al., 2023). SUMOylation of METTL3 reduces its m^6^A methytransferase activity, hence decreasing m^6^A levels in mRNAs (Du et al., 2018). The precise function of METTL16 is still being explored particularly with respect to its roles in mRNA and snRNA methylation (Satterwhite and Mansfield, 2022). In addition, it has been shown to significantly affect various cellular processes (Talic et al., 2023). WTAP stabilizes the core complex and promotes METTL3-METTL14 heterodimer to the nuclear speckles. The RBM15/15B is essential in assisting binding of METTL3 and WTAP, directing the two proteins to their target sites. VIRMA directs the methyltransferase components to specific RNA regions. Other proteins, such as ZC3H13 and CBLL1, together with additional cofactors, including WTAP, regulate nuclear m^6^A methylation (Zhou et al., 2020; Jiang et al., 2021).

The m^6^A mRNA modifications are installed to nascent pre-mRNA molecules and chemical modification is finished by the release of mRNA into nucleoplasm. Moreover, quantitative m^6^A analysis suggested that little of the methylation reaction actually takes place in the cytoplasm (Ke et al., 2017). The m^6^A modification affects multiple stages of the mRNA life cycle such as splicing, nuclear export, translation, and degradation (Bedi et al., 2023) and altered m^6^A levels disturb gene expression and other essential cellular processes (Zhou et al., 2020). Regarding the role of m^6^A modification in mRNA splicing, it is important to note that a study suggested that m^6^A mRNA modifications are not essential for most splicing events (Ke et al., 2017). The m^6^A RNA modification is a reversible reaction. The m^6^A can be removed by RNA demethylases. At present, two RNA demethylases are known, fat mass and obesity-associated protein (FTO) and alkylation protein AlkB homolog 5 (ALKBH5) (Shen et al., 2022). The FTO was first discovered to exhibit demethylase activity to m^6^A in 2011 (Jia et al., 2011). The ALKBH5 was first identified in 2013 (Zheng et al., 2013). Both demethylases and methyltransferases collectively contribute to the modulation of m^6^A levels in eukaryotic organisms (Shen et al., 2022). Of note, the role of FTO as a demethylase for m^6^A and m^6^Am or for m^6^Am only, remains ambiguous (Nabeel-Shah et al., 2024).

### 3.2 N6,2-O-dimethyladenosine (m^6^Am)

Beside modification to form m^6^A, the adenine base of RNA molecule can undergo alteration to generate m^6^Am, m^1^A, and can be edited to inosine (A-to-I) (Adamopoulos et al., 2023; Wu et al., 2023b). The m^6^Am is resulted from adenosine N6-methylation of 2′-O-methyladenosine (Am) (Mauer et al., 2019). In the case that the first nucleotide after the m^7^G cap is adenosine, it will be methylated at the N6-position to form m^6^Am catalyzed by an enzyme called phosphorylated C-terminal domain (CTD) interacting factor 1 (PCIF1) (Wei et al., 1975; Akichika et al., 2019; Sendinc et al., 2019). The majority of PCIF1 is found in the nucleus, playing a role in generating the m^6^Am modification on new transcripts. Currently, PCIF1 is the only mammalian methyltransferase of m^6^Am known (Sendinc et al., 2019; Wu et al., 2023b). Similar with m^6^A RNA modification, the m^6^Am modification is also a reversible reaction. It is dynamically modulated by PCIF1 and FTO (Sun et al., 2021). The FTO RNA demethylase functions as an eraser which removes the methyl group from the N6-position (Cesaro et al., 2023).

### 3.3 N1-methyladenosine (m^1^A)

The m^1^A modifications have been found in tRNA, rRNA, mRNA, and mitochondrial tRNA (Jin et al., 2022; Adamopoulos et al., 2023). The m^1^A RNA modification is catalyzed by TRMT10 and the TRMT6/TRMT61 complex. The protein subunits of this complex are members of tRNA methyltransferase (TRMT) protein family. Similarly to the m^6^A RNA modification, the m^1^A RNA modification is reversible in nature. The m^1^A chemical modification can be removed by ALKBH1 and ALKBH3, the key enzymes functioning as erasers for this type of RNA modification (Adamopoulos et al., 2023). Readers for m^1^A include YTHDC1, YTHDF2, and YTHDF3 (Adamopoulos et al., 2023).

### 3.4 Adenosine-to-inosine (A-to-I)

A-to-I RNA editing is one of the most common posttranscriptional RNA modifications in metazoans and in humans (Yang Y. et al., 2021; Adamopoulos et al., 2023). This base conversion reaction is catalyzed by enzymes termed adenosine deaminases acting on RNA (ADARs). These enzymes (ADAR1 and ADAR2) are present throughout the body but are most abundant in the central nervous system (Slotkin and Nishikura, 2013). The A-to-I editing has been found in both coding and ncRNA transcripts (Yang Y. et al., 2021). As the translation machinery generally interprets inosine as guanosine, A-to-I editing within the coding sequence can cause amino acid substitution and diversify the proteome (Gabay et al., 2022). Different from other RNA modifications such as RNA methylation, the A-to-I editing process is totally regulated by ADARs without the involvement of other readers or erasers (Li et al., 2021).

### 3.5 5-Methylcytidine (m^5^C)

In addition to adenine, the cytosine of RNA molecule can also undergo posttranscription modifications. Cytosine can be modified to generate m^5^C and 3-methylcytosine (m^3^C). The cytosine base can also be edited to form uridine (C-to-U RNA editing) (Adamopoulos et al., 2023). The m^5^C RNA modification is catalyzed by RNA m^5^C methyltransferases (RCMTs), which consist of the NOL1/NOP2/SUN domain (NSUN) family of proteins and DNA methyltransferase (DNMT) homologue DNMT2 (Gao and Fang, 2021; Li M. et al., 2022). The m^5^C RNA modification is a reversible reaction. The removal of m^5^C is catalyzed by enzymes termed the “ten-eleven translocation” (TET) family proteins which oxidize m^5^C in RNA into cytosine-5-hydroxymethylation (hm^5^C) (Gao and Fang, 2021). Two proteins have been identified as readers for m^5^C, YBX1 and ALYREF (Gao and Fang, 2021).

### 3.6 5-Hydroxymethyl cytidine (hm^5^C)

In mammals, m^5^C can undergo oxidative processing generating hm^5^C and 5-formylcytidine (f^5^C) (Huber et al., 2015). The hm^5^C has been identified in all three domains of life, and is mainly present in mRNA (Huber et al., 2015; Huang et al., 2016). The TET enzymes which catalyze oxidative demethylation of m^5^C in DNA molecule forming hm^5^C, were also found to catalyze formation of hm^5^C in human cells *in vitro* (Fu et al., 2014). A study using *Drosophila melanogaster* demonstrated that hm^5^C is deposited by TET methyldioxygenases. Furthermore, TET and hydroxymethylated RNA were found to be the most abundant in the *Drosophila* brain. Of note, hm^5^C also occurs, and is well documented, in DNA (Delatte et al., 2016). The hm^5^C RNA modification was also found in mouse brain but at a lower level than for the hm^5^C DNA modification (Miao et al., 2016).

### 3.7 3-Methylcytidine (m^3^C)

The m^3^C RNA modification has been identified in both tRNA and mRNA (Xu et al., 2017; Chen et al., 2021). It was reported that in eukaryotic cells the m^3^C RNA modification is widely distributed at position C32 of tRNAThr and tRNASer molecules (Mao et al., 2021). The cellular formation of m^3^C is catalyzed by the writer enzymes RNA methyltransferases. Notably, RNA methyltransferases constitute a diverse family of enzymes that transfer a methyl group from SAM to a variety of positions in RNA. There are currently 4 METTL enzymes (METTL2A, METTL2B, METTL6, and METTL8) found in mammals (Mao et al., 2021; Lentini et al., 2022). The METTL8 is responsible for catalyzing the m^3^C addition in human mitochondrial tRNAs (Lentini et al., 2022). Of note, only two methyltransferases (Trm140 and Trm141) were identified in fission yeast and only one (Trm140) was present in budding yeast (Mao et al., 2021). The m^3^C RNA modification is reversible. There are two demethylases (erasers), ALKBH1 and ALKBH3 which have been identified in human cells. ALKBH1 removes methyl (CH3) groups in human mRNA (Ma et al., 2019) while ALKBH3 demethylates human tRNA. It should be noted that ALKBH3 is also a m^1^A demethylase of tRNA (Chen Z. et al., 2019).

### 3.8 Cytidine to uridine (C-to-U)

The C-to-U RNA editing has been found in both mammals and plants. The molecular mechanism of C-to-U RNA editing involves the hydrolytic deamination of a cytosine to a uracil base which is catalyzed by multiple cytosine deaminases, which belong to a family of mammalian enzymes known as the “activation-induced cytidine deaminase/apolipoprotein B mRNA-editing enzyme catalytic polypeptide-like” (AID/APOBEC) protein family. The first member of this family is APOBEC1 (Pecori et al., 2022). The activity of RNA-specific cytidine deaminases requires several complementation factors (Vu and Tsukahara, 2017). The C-to-U RNA editing is subject to induction by relevant environmental factors such as hypoxia (Baysal et al., 2013). This editing may alter the characteristics of the encoded proteins. For example, the C-to-U editing in the nuclear transcript encoding intestinal apolipoprotein B (apoB) resulted in a truncated apoB protein. This editing reaction is catalyzed by APOBEC1 cytidine deaminase which changes a CAA to a UAA stop codon (Blanc and Davidson, 2003; Baysal et al., 2013). Similarly, the C-to-U RNA editing which changes an arginine (CGA) to a UGA translational stop codon, in the neurofibromatosis type 1 (NF1) mRNA in mammals, is predicted to generate a truncated neurofibromin protein. Of note, neurofibromin is a large and multifunctional protein encoded by the tumor suppressor gene *NF1* (Mukhopadhyay et al., 2002; Baysal et al., 2013). Overexpression of exogenous APOBEC3A was reported to induce C-to-U RNA editing of thousands of genes (Sharma et al., 2017).

### 3.9 Pseudouridine (Ψ)

Ψ is a derivative of uridine (U) formed via base-specific isomerization reactions catalyzed by pseudouridine synthases (PUSs). There are 13 PUSs found in humans (Borchardt et al., 2020). Ψ is found in both ncRNA and mRNA and is conserved across species (Zhao and He, 2015). There are two independent molecular mechanisms underlying the formation of Ψ. The first mechanism involves single protein enzymes (PUSs), which recognize the substrate and catalyze the isomerization of uridine to Ψ (RNA-independent pseudouridylation). In contrast, the second mechanism is an RNA-dependent mechanism involving unique RNA and four common core proteins. The RNA component functions as a guide which base pairs with the substrate RNA and directs an enzyme (Cbf5), which is part of the core proteins, to carry out the pseudouridylation reaction at a specific site (De Zoysa and Yu, 2017). Unlike m^6^A, m^6^Am, and m^1^A, which are all reversible, the conversion from U to Ψ is irreversible (Zhao and He, 2015). Notably, compared to uridine, Ψ has an extra hydrogen-bond donor at its non-Watson-Crick edge. Therefore, when incorporated into RNA, it can change the chemical and physical properties of RNA and hence its cellular function (Zhao and He, 2015).

### 3.10 2′-O-methylation (Nm)

RNA 2′-O methylation (Nm, where N stands for any nucleotide) is a common RNA modification found in different types of RNA such as rRNA, tRNA, mRNA, and sncRNAs (miRNAs and siRNAs). The Nm modification is generated by addition of a methyl group to 2′ hydroxyl (–OH) of the ribose component of nucleotide either co- or post-transcriptionally (Dimitrova et al., 2019). It is catalyzed by either stand-alone methyltransferases or by the enzyme fibrillarin which is guided by snoRNAs. It has been suggested that Nm RNA modification may cause structural bias which leads to a more stable RNAs and alter cellular activities of the RNA molecules (Abou Assi et al., 2020). It was found that inflammation promotes secretion of snoRNA out of the nucleus, and RNA-Seq data indicate that extracellular vesicles released from cells harbor snoRNAs. These suggest the extended role of snoRNA in cell-cell communication (Rimer et al., 2018). Most mammalian mRNAs have 2′-O methylation at nucleotide 1 (cap 1 mRNA) (Bélanger et al., 2010).

### 3.11 N7-methylguanosine (m^7^G)

M^7^G is a common RNA modification which occurs at the 5′ terminal (m^7^G-cap) or within RNA molecules. The m^7^G has been found in tRNA, rRNA, mRNA, and miRNA (Chu et al., 2018; Luo et al., 2022; Cai et al., 2023). Different m^7^G methyltransferases (writers) have been identified. In mammals, METTL1, which binds to its cofactor WD repeat domain 4 (WDR4), catalyzes m^7^G modifications in tRNA, miRNA, and mRNA. Internal m^7^G is recognized by Quaking proteins (QKIs) which also bind to the stress granule (SG) core protein G3BP1 thereby recruiting internal m^7^G-modified transcripts into SGs presumably to regulate their stability and translation (Zhao et al., 2023).

## 4 Cap modification

### 4.1 Canonical RNA capping

**FIGURE 2:**
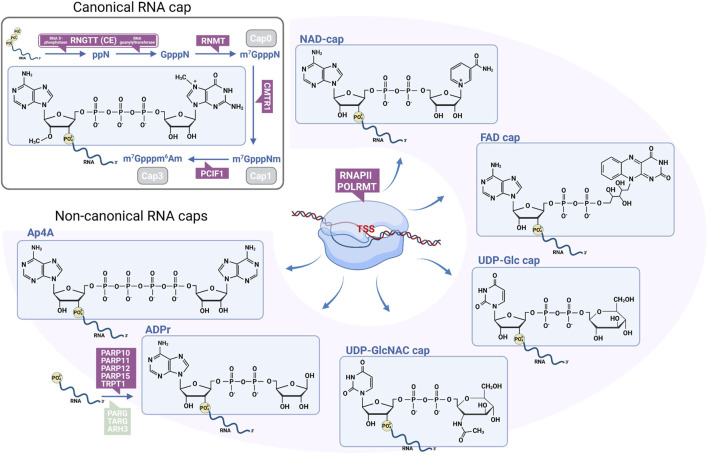
Canonical and non-canonical RNA 5′ cap structures. The canonical pathway requires the RNA 5′-phosphatase activity of RNGTT to cleave the γ-phosphate from the nascent mRNA. Next, the guanylyltransferase domain of RNGTT transfers a GMP through its 5′ phosphate to the 5′ diphospho-RNA. RNMT then methylates the N7 position of the terminal guanine from SAM. The m^7^GpppN structure (Cap 0) can be further methylated by CMTR1 to form m^7^GpppNm (Cap 1) and by CMTR2 to form m^7^GpppNmNm (Cap 2, not shown) structures. When the penultimate nucleoside is adenosine, PCIF1 methylates the N6 position of the adenosine to form m^7^Gpppm^6^Am (Cap 3). The non-canonical caps are generated by RNAPII or POLRMT when they initiate transcription with an adenine or uridine nucleotide-containing metabolite instead of ATP or UTP. ADPr-caps can also be generated by several PARPs or TRPT1 through direct ADP-ribosylation of the 5′-phoshate end of an RNA. While the ^7^mG-cap stabilizes the mRNA and is essential for efficient translation, non-canonical capped RNAs are not transcribed and have varying effect on stability. However, the RNAs capped in this way are protected from innate immune recognition within the cell. ADPr, ADP-ribose; Ap4A, diadenosine tetraphosphate; ARH, ADP-ribosylserine hydrolase; CE, capping enzyme; CMTR, cap (nucleoside-2′-O)-methyltransferase; FAD, flavine adenine dinucleotide; NAD, nicotinamide adenine dinucleotide; PARP, poly(ADP-ribose)-polymerase; PARG, poly(ADP-ribose) glycohydrolase; PCIF1, phosphorylated CTD interacting factor 1; POLRMT, mitochondrial RNA polymerase; RNAPII, RNA polymerase II; RNGTT, RNA guanilyltransferase; RNMT, RNA (guanine N7)-methyltransferase; RTPase, RNA triphosphatase; SAM, S-adenosyl-L-methionine; TARG, O-acyl-ADP-ribose deacylase; TRPT1, tRNA phosphotransferase 1; TSS, transcription start site; UDP-Glc, uridine diphosphate glucose; UDP-GlcNAM, uridine diphosphate N-acetylglucoseamine. Created in BioRender. Demeny (2025) https://BioRender.com/l07c373.

The account of RNA modifications would be incomplete without briefly addressing the modifications of the RNA ends (Figure 2). Except for circRNA, cellular RNA molecules are linear polymers with 5′ and 3′ ends. These ends are potentially vulnerable to degradation by exonucleases or recognition by innate immune sensors like RIG-I, MDA5, or IFITs mediating defense against intracellular bacteria and viruses, whence they must be protected (Leung and Amarasinghe, 2016). Long before the internal modifications, it was discovered that most eukaryotic cellular mRNAs carry a 5’ “cap,” m^7^GpppN that protects the mRNA against attack by phosphatases and nucleases. 5′-mRNA capping occurs shortly after and in concert with transcription initiation. The 5′-capping enzymes, RNA guanylyltransferase (RNGTT) (harboring both 5′-triphosphatase and guanylyltransferase activities) and RNMT, are targeted to the pre-mRNA through binding to the phosphorylated carboxy-terminal domain of RNA polymerase II (RNAPII) (Figure 2). In higher eukaryotes, the m^7^GpppN structure (cap 0) can be methylated also at the ribose’s 2′-O position within the second (cap 1) and third (cap 2) nucleotides by the cap methyltransferases 1 and 2 (CMTR1 and 2) (Bélanger et al., 2010; Werner et al., 2011). A subset of RNAP II-transcribed cellular RNAs, including snRNA, snoRNA, and telomerase RNA, are further methylated at the N2 of the guanosine to create an trimethylguanosine (m2,2,7G)-capped RNA (Monecke et al., 2009). Besides stabilizing the RNA, the cap has been shown to facilitate splicing, nuclear export, and translation initiation by recruiting protein complexes involved in RNA processing (Shatkin, 1976; Rottman et al., 1974; Hamm and Mattaj, 1990; Izaurralde et al., 1994). The splicing and nuclear export-related effects of RNA capping can be ascribed to the cap-binding complex, CBC. The RNA-binding subunit CBP20 forms CBC with its partner, CBP80. CBC also mediates RNA quality control in the nonsense-mediated decay pathway (Schoenberg and Maquat, 2012). The primary reader for the m^7^G cap modification during translation is eukaryotic translation initiation factor 4E (EIF4E).

### 4.2 Non-canonical RNA capping

During the past decade, novel metabolite-derived (NAD, FAD, ADPr, dpCoA, UDP-Glc, and UDP-GlcNAC) terminal cap structures have been discovered, the biosynthesis of which differs from that of the m^7^G-cap (Pelletier et al., 2021; Wiedermannová et al., 2021) (Figure 2). Metabolite caps are incorporated into the RNA when an RNAP initiates transcription with an adenine nucleotide-derived cofactor or an UDP-sugar instead of an ATP or UTP at transcription start sites featuring A or U in the +1 position (Julius and Yuzenkova, 2017). These non-canonical initiating nucleotide (NCIN) caps have been found in mRNAs of nuclear and mitochondrial origin, snRNAs, and snoRNAs. In mammals, the frequency of metabolite caps is 0.1%–5% and up to 15% in mitochondrial RNA, determined by an interplay between the metabolites’ availability, the RNA polymerases’ affinity, and transcript-specific promoter sequences (Wang et al., 2019). Dinucleoside polyphosphates (NpnN), also called alarmones, are stress-related molecules in bacteria and eukaryotes, although their function is not precisely understood. Diadenosine tetraphosphate (Ap4A), the most abundant NpnN in humans, can also be incorporated into RNA by RNAPII as an NCIN (František Potužník et al., 2024). Interestingly, for this modification, the amount of modified RNAs appears to be independent of the abundance of Ap4A in the cell.

#### 4.2.1 RNA regulation by non-canonical caps

NCIN-capped mRNAs are generally not translated in human cells, but the various caps have been shown to confer different stability. In eukaryotes, the NAD-cap was found to promote RNA decay by the decapping exoribonuclease (DXO) and the Nudix hydrolases, Nudt12 and Nudt16, whereas Ap4A-capped RNA is as stable as a canonical m^7^G-RNA (Jiao et al., 2017; František Potužník et al., 2024). Various forms of cellular stress have been shown to increase the abundance of NAD-capped RNAs, establishing a link between the cell’s metabolic state, redox homeostasis, and post-transcriptional RNA regulation (Grudzien-Nogalska et al., 2019). Regulation of the level of decapping enzymes under these conditions suggests the further possibility that once the stress has subsided, the cell may revert from NCIN caps to canonical caps through cap-removal and recapping (Grudzien-Nogalska et al., 2019). Cytoplasmic addition of an m^7^GpppN cap to uncapped RNA is mediated by RNGTT, which has been shown to translocate from the nucleus to the cytoplasm and to form a complex there with RNMT, the latter’s regulatory subunit RAM, and an, as yet, unidentified RNA 5′-monophosphate kinase (Otsuka et al., 2009; Trotman et al., 2017; Gonatopoulos-Pournatzis et al., 2011). Nck-1, a scaffold protein that binds to the proline-rich C-terminus of RNGTT is also a component of the cytoplasmic capping enzyme complex, and it is known to help restore translation following stress by directly interacting with eIF2 and blocking its phosphorylation (Mukherjee et al., 2014). The recently reported association of the cytoplasmic capping enzyme with SGs – membrane-less organelles regarded as translation regulation hubs during stress – equally supports the idea that the cap status of non-canonically capped translationally suppressed transcripts may be restored (Gayen et al., 2024; Baymiller and Moon, 2023).

## 5 Role of RNA modification in gene expression

Regulation of gene expression is critical for a wide variety of key biological processes, such as organismal development, cell differentiation, cellular stress responses, tissue homeostasis, and immunity (Pope and Medzhitov, 2018). RNA modifications serve as critical posttranscriptional regulators of gene expression programs and their correct deposition is essential for normal development (Frye et al., 2018). Accumulating evidence reveals that the dynamics of internal RNA modifications play critical roles in multiple RNA-processing events including splicing, transport, translation, and degradation all of which in turn regulate gene expression (Zhao et al., 2017; Dominissini and Rechavi, 2018). The expression patterns of RNA modifications and also of their regulators have the potential to be used as biomarkers for diseases or absorption of disease-causing hazard (Chen et al., 2022; Chen et al., 2023; Takahashi et al., 2023). There is mounting evidence that RNA modifications are associated with diverse biological processes including human diseases. Mutations of the genes encoding RNA modifying enzymes have been linked to basic cellular functions such as cell differentiation, sex determination, stress responses, and various human diseases including cancer, cardiovascular diseases, genetic birth defects, metabolic diseases, neurological disorders, and mitochondrial-related defects (Jonkhout et al., 2017). Due to the recent intensive research, a large amount of relevant published reports has appeared. Only selected articles are included in the following discussion on the role of RNA modification in gene expression.

The m^6^A is known to affect various fundamental cellular processes by regulating target gene expression (Liu and Pan, 2016). In modulating gene expression, m^6^A controls mRNA stability (Wang et al., 2014), translation efficiency (Wang et al., 2015), and RNA-protein interactions (Liu et al., 2015). The m^6^A RNA modifications have been linked to various diseases and deterioration of physiological functions such as pancreatic carcinoma (Cao et al., 2023), hepatocellular carcinoma (HCC) (Liu H. et al., 2023), ovarian cancer (Gan et al., 2023), glioma (Wu Z. et al., 2023), osteoarthritis (Liu Y. et al., 2023), Alzheimer’s disease (Ni et al., 2023), pulpitis (Xu et al., 2023), metabolic disorder (Liu K. et al., 2023), impaired immunity (Zhang Y. et al., 2023), hearing loss (Feng et al., 2023), hypoxia (Li S. et al., 2023), aging (Huang et al., 2023), male infertility (Li H. et al., 2023), viral infection (Vaid et al., 2023), *etc*. The m^6^A mRNA methylase, WTAP, has been demonstrated to promote progression of diffuse large B-cell lymphoma (DLBCL) by inducing the expression of its target gene hexokinase 2 (HK2), hence increasing the HK2 m^6^A level. Of note, DLBCL is one of the most common subtypes of lymphoid malignancy (Han et al., 2021). The m^6^A regulators may serve as a prognostic signature for esophageal squamous cell carcinoma (ESCC). As many as six m^6^A regulators, METTL3, WTAP, IGF2BP3, YTHDF1, HNRNPA2B1, and HNRNPC, showed increased expression in patients with ESCC. Similarly, increased expression of programmed cell death ligand 1 (PD-L1) was also observed. It was suggested that the m^6^A methylation regulators play a key role as a mediator for PD-L1 expression (Guo et al., 2021). YTHDF1 has also been reported to promote ovarian cancer progression by augmenting translation of EIF3C, a subunit of eukaryotic initiation factor 3 (EIF3), a complex translation initiation factor in mammalian cells (Liu T. et al., 2020).

Overexpression of a newly discovered m^6^A reader, named IGF2BP2, was shown to promote lymphatic metastasis and epithelial mesenchymal transition of head and neck squamous carcinoma (HNSCC) through stabilization of mRNA in an m^6^A-dependent fashion. Notably, epithelial mesenchymal transition is a process by which epithelial cells gain migratory and invasive properties. Overexpression of IGF2BP2 was demonstrated to be associated with a poor overall survival probability of patients with HNSCC (Yu et al., 2022). Following their discovery, more and more studies have been conducted to elucidate the physiological functions of m^6^A erasers, FTO and ALKBH5, and their roles in disease development (Shen et al., 2022). The association between the demethylase FTO and obesity is currently well documented (Tóth et al., 2020; Al-Jawadi et al., 2021; Vámos et al., 2023), while ALKBH5 has been indicated to play a role in human malignancies (Qu et al., 2022). Recently, it was reported that m^6^A mediates expression of Frizzled 10 (FZD10) in liver cancer stem cells (CSCs), which in turn stimulates FZD10 self-renewal, tumorigenicity, and metastasis of liver CSCs. The METTL3-dependent m^6^A modification of FZD10 mRNA also leads to lenvatinib resistance of the CSCs (Wang et al., 2023a). In addition, a study showed that m^6^A modification of eRNA leads to its activation and promotes transcription and gene activation (Lee et al., 2021).

It has been suggested that m^6^Am exhibits a significant impact on gene expression regulation. The specific methyltransferase of m^6^Am, PCIF1, has been indicated to affect mRNA stability, transcription, and translation. Moreover, PCIF1 has been associated with tumor, viral, and endocrine diseases (Wu et al., 2023b). The m^6^Am modification has unequivocally demonstrated to increase mRNA stability, translation efficiency, and protein levels which may play a dynamic role in obesity-related translation regulation (Ben-Haim et al., 2021). The m^6^Am RNA modification was suggested to have a negative impact on the translation of mRNAs with m^6^Am at the 5′ end (Sendinc et al., 2019). However, a study has recently demonstrated that the PCIF1-mediated installment of 5′-cap m^6^Am increases susceptibility to severe acute respiratory syndrome coronavirus 2 (SARS-CoV-2) infection by stabilizing mRNA which leads to sustained transcription and translation of genes encoding the coronavirus receptors angiotensin-converting enzyme 2 (ACE2) and transmembrane serine protease 2 (TMPRSS2) (Wang L. et al., 2023).

A-to-I RNA editing is important to prevent undesired immune activation (Mann et al., 2023). In addition, dysregulation of A-to-I RNA editing has also been associated with neurological or neurodegenerative diseases such as amyotrophic lateral sclerosis, epilepsy, depression, encephalopathy, suicidal behavior associated with schizophrenia, astrocytoma, bipolar disorder, and episodic ataxia type 1 (Yang Y. et al., 2021). In addition, aberrance in ADAR activity has been linked to human diseases such as cancer, metabolic diseases, viral infections, and autoimmune disorders (Slotkin and Nishikura, 2013). Increased A-to-I RNA editing was observed in relapsed tumor samples from patients with melanoma during targeted therapy. This may be due to increased expression of ADAR enzymes because RNA editing indexes showed positive correlation with the expression levels of genes coding for ADAR enzymes (Amweg et al., 2022). Similarly, A-to-I RNA editing was shown to play a critical role in the development of liver cancer. It was found that in tumor samples, expression of the gene encoding the enzyme ADAR was elevated and A-to-I RNA editing was enhanced. In addition, it was indicated that ADAR regulates its own expression by self-editing, and also affects the global transcription and translation products of cancer-related genes by editing and changing their expression profiles (Li et al., 2021). Recently, a study reported increased A-to-I RNA editing in patients with atherosclerosis, cardiomyopathies, and heart failure. The insulin-like growth factor binding protein 7 (IGFBP7) was identified as the main editing site. Of note, IGFBP7 is a protein which functions to regulate the availability of insulin-like growth factors and their binding to their receptors (Mann et al., 2023). The A-to-I RNA editing events were suggested to be involved in Parkinson’s disease through their effects on gene expression. The editing events were found to occur mainly in protein-coding genes and Arthrobacter luteus (Alu) repeats. Lower overall editing frequency, and hence, decreased editing levels were observed in patients with Parkinson’s disease. It was proposed that A-to-I RNA editing regulates gene expression by changing the miRNA binding sites of the host gene (Wu S. et al., 2023). A study using *Caenorhabditis elegans* demonstrated that A-to-I RNA editing stimulates developmental stage–specific genes and the expression of lncRNA. As competition between RNA editing mechanisms and RNA interference (RNAi) had previously been indicated, it was hypothesized that A-to-I RNA editing is essential for normal growth and development by regulating the process of silencing gene expression through RNAi (Goldstein et al., 2017).

The m^5^C has been identified in mRNA, rRNA, and tRNA in organisms from all species and plays a critical role in diverse biological processes such as the modulation of transcription, RNA stability, and protein synthesis (Song et al., 2022). The m^5^C reader protein, YBX1 is essential for mediating mRNA stability (Chen X. et al., 2019) and the reader, ALYREF plays a role in facilitating mRNA nuclear export (Dominissini and Rechavi, 2017; Yang et al., 2017). The m^5^C methyltransferase, NSUN6 was indicated to suppress pancreatic cancer development by controlling cell proliferation. Significantly reduced expression of NSUN6 was observed in pancreatic cancer tissues compared to normal controls (Yang R. et al., 2021). Overexpression of m^5^C methyltransferase, NSUN2 has been found to cause resistance of small-cell lung cancer to the epidermal growth factor receptor (EGFR) inhibitor, gefitinib. The mechanism was suggested to involve increased methylation of the quiescin sulfhydryl oxidase 1 (QSOX1) coding sequence region which leads to enhanced QSOX1 translation through m^5^C reader Y-box binding protein 1 (YBX1) (Wang Y. et al., 2023). The m^5^C RNA modification was also found to play a role in regulating the innate immune response to virus infection by modulating type I interferons. Depletion of m^5^C methyltransferase, NSUN2, was demonstrated to reduce m^5^C methylation and inhibit replication and gene expression of different viruses, although the m^5^C methylation of viral RNA was unaffected (Zhang et al., 2022). The m^5^C has been found to activate cancer metastasis by promoting mitochondrial protein translation. In mitochondria, the biosynthesis of the mitochondrially encoded subunits of the oxidative phosphorylation complexes is dependent on formation of m^5^C at position 34 in the mitochondrial methionine tRNA. Notably, the mitochondrial oxidative phosphorylation system plays a critical role in the efficient generation of cellular energy in the form of ATP. A metabolic switch from glycolysis to oxidative phosphorylation was found to facilitate tumorigenesis (Delaunay et al., 2022).

The hm^5^C RNA modification has been indicated to play an important regulatory role inside the cells (Huber et al., 2015). In mammals, TET2 was reported to stimulate pathogen infection-induced myelopoiesis, a common host immune response in acute and chronic infections (Shen et al., 2018). A study employing mouse embryonic stem cells, suggested that hm^5^C plays an important role in the regulation of the embryonic stem cell self-renewal network. In this study, Tet mediated RNA hydroxymethylation was found to reduce the stability of pluripotency promoting transcripts. A reduced level of hm^5^C was observed during cell differentiation. It was hypothesized that hm^5^C is a mark of transcriptome flexibility which is important for controlling the balance between pluripotency and lineage commitment (Lan et al., 2020). In *Drosophila melanogaster*, it was discovered that RNA hydroxymethylation promotes RNA translation. As previously mentioned, Tet and hm^5^C were prevalent in *Drosophila* brain. Fruit flies lacking Tet suffer from decreased RNA hydroxymethylation and impaired brain development (Delatte et al., 2016).

The detailed biological function of m^3^C RNA modification has yet to be fully elucidated. Considering that it is mainly present in the anticodon loop of tRNAs, it is hypothesized that m^3^C affects precise pairing between codon and anti-codon (Mao et al., 2021). It has also been indicated that m^3^C is important for tRNA structure and folding (Lentini et al., 2022). The m^3^C RNA modification has been suggested to be essential for ensuring proper architecture of tRNAs which is critical for translation fidelity. The lack of tRNA m^3^C modifications may cause impaired translation process (Bohnsack et al., 2022).

Dysregulation of C-to-U miRNA editing may contribute to pathogenesis of Huntington’s disease (Guo et al., 2022). A study suggested that APOBEC3-mediated C-to-U RNA editing is positively associated with elevated immune activity and improved survival of patients with breast cancer (Asaoka et al., 2019). Mutations in the APOBEC1 cofactors, RBM47, have been linked to breast cancer progression and increased metastatic potential (Lerner et al., 2019). C-to-U RNA editing has been indicated to accelerate the evolution of RNA viruses such as SARS-CoV-2. Comparative genomic analysis of world-wide SARS-CoV-2 strains showed that C-to-U RNA editing is the main source of SARS-CoV-2 mutation (Wang et al., 2023b).

RNA pseudouridylation has been suggested to affect RNA metabolism and gene expression (Borchardt et al., 2020). In humans, co-transcriptional pseudouridylation of pre-mRNA was found to be essential for pre-mRNA processing. Three PUSs, PUS1, PUS7, and RNA PUS D4 (RPUSD4), were suggested to be involved in pseudouridylation process (Martinez et al., 2022). Ψ has been indicated to increase transcript stability (Schwartz et al., 2014) and therefore it can alter efficiency of translation initiation and other cellular processes (Carlile et al., 2014). Similarly, a study found that in yeast, pseudouridylation of tRNA and mRNA by PUS6 is essential for promoting translation. The mechanism involves increased binding of yeast methionine aminoacyl tRNAMet synthetase (MetRS), which functions as a reader, to both pseudouridylated tRNA and pseudouridylated mRNA which results in an enhanced translation process (Levi and Arava, 2021). A previous study has also demonstrated that when uridine molecules in the mRNA are replaced with Ψ, the translation level is improved. The mechanism involves decreased activation of RNA-dependent protein kinase (PKR), a mammalian enzyme which regulates translation during stress conditions (Anderson et al., 2010). In humans, mutations in PUS3 protein were shown to reduce PUS3-dependent Ψ levels which cause intellectual disability (Lin et al., 2022). Similarly, mutations in human PUS7 were found to cause intellectual disability and microcephaly due to impaired pseudouridylation (Shaheen et al., 2019). Ψ is also installed to RNA molecules by the H/ACA small ribonucleoprotein (snoRNP) complex which shares four core proteins, dyskerin (DKC1), NOP10, NHP2, and GAR1. It was reported that mutations in *DKC1* and *NOP10* genes cause nephrotic syndrome with cataracts, hearing impairment, and enterocolitis (Balogh et al., 2020). Recently, DKC1 was indicated to play a role in regulating translation via mRNA pseudouridylation (Pederiva et al., 2023). The translation process was also shown to be regulated by rRNA pseudouridylation (Zhao et al., 2023). Furthermore, alterations of rRNA pseudouridylation levels at specific sites have been linked to human breast cancer (Barozzi et al., 2023).

In human cells, the snoRNA-guided Nm modifications of mRNA have been suggested to play an important role in modulating gene expression by altering mRNA levels and controlling protein biosynthesis. Nm RNA modifications were found to increase peroxidasin mRNA expression but inhibit its translation (Elliott et al., 2019). Similarly, in yeast, Nm RNA modifications were also suggested to play a role in translation regulation. Aberrant rRNA Nm patterns or hypo-2′-O-methylated ribosomes were revealed to cause drastic defects in translation fidelity (Khoshnevis et al., 2022). The expression of the Nm factors which mediate RNA 2′-O-methylation was shown to be associated with malignant melanoma formation. Upregulation of Nm factors such as fibrillarin, nucleolar protein (NOP) 56, NOP58, or SNU13 was found to be correlated with this disease and has a negative impact on overall survival of patients with melanoma (Jasinski-Bergner et al., 2021). Nm within bacterial RNA was suggested to suppress activation of human innate immune response by inhibiting Toll-like receptor (TLR) 7-mediated-IFN-α production. It is important to note that the innate immune system plays a critical role in the early sensing and clearance of infecting pathogens and differences in posttranscriptional RNA modification profiles are used by the immune system to discriminate between the host and pathogenic nucleic acids. This principle is likely exploited by certain bacteria to evade the host immune responses (Rimbach et al., 2015). Increased Nm was observed in polyadenylated RNA in virus infected-macrophages. Fibrillarin and its mediated Nm RNA modifications may promote viral infection (Li P. et al., 2022). The internal Nm RNA modifications on the human immunodeficiency virus type-1 (HIV-1) genome are employed by the virus to limit the host immune sensing and interferon production. However, the Nm marks are observed to impair HIV-1 reverse transcriptase activity and hence inhibit viral replication (Decombe et al., 2024). The human mRNA Cap 2′-O-Methyltransferase 1 (CMTR1) was found to regulate the expression of certain interferon-stimulated genes which are essential for restricting viral infection. CMTR1 was shown to mediate the protein expression of IFN-stimulated genes by preventing interferon-induced protein with tetratricopeptide repeats 1 (IFIT1) from inhibiting the translation of mRNAs lacking cap 2′-O-methylation. Therefore, CMTR1 stimulates the IFN-mediated antiviral response (Williams et al., 2020).

The m^7^G modification is associated with the biological processes and regulation of various diseases. It has been indicated that m^7^G is tightly linked to tumor prognosis, development, and the immune response. A number of m^7^G regulatory genes have been proposed as risk signatures of HCC considering their significant effects on prognosis, progression, and antitumor immune response of HCC (Zhou et al., 2022). A study has indicated that m^7^G-related lncRNAs are associated with the tumor immune landscape and the prognosis of HCC. Moreover, as many as 32 m^7^G-related lncRNAs were confirmed to be prognostic lncRNAs and can be applied as independent prognostic markers of HCC (Li Y. et al., 2023). Similarly, expression of several m^7^G methylation-related regulator genes, such as *EIF4E3*, *LARP1*, *NCBP3*, and *IFIT5* have been shown as good prognostic predictors for melanoma (Deng et al., 2022). Recently, a study revealed the involvement of m^7^G in the development of drug resistance in acute myeloid leukemia (AML), a type of blood cancer characterized by uncontrolled proliferation of myeloid cells. It was observed that lncRNA m^7^G methylations are more abundant in drug-resistant AML cells compared to that in drug-sensitive AML cells (Han et al., 2023). Similarly, an association between m^7^G modifications in circRNAs and drug-resistant AML has been suggested. A significant difference in m^7^G level between AML cells and drug-resistant AML cells was found which indicates a potential role of m^7^G in circRNAs in drug-resistant AML development. It was hypothesized that the m^7^G methylation affects co-expression of circRNA, miRNA, and mRNA which may further affect the modulation of resistance-associated genes in AML (Fu et al., 2023). Recently, it has been reported that internal m^7^G when located within a GANGAN (N = A/C/U/G) motif is selectively recognized by QKIs (Zhao et al., 2023). QKIs transport internal m^7^G-modified RNAs into SGs presumably modulating the modified mRNA’s half-life and expression. QKI7, e.g., attenuates the translation efficiency of essential genes in the Hippo signaling pathway sensitizing cancer cells to chemotherapy.

## 6 Therapeutic developments

**FIGURE 3:**
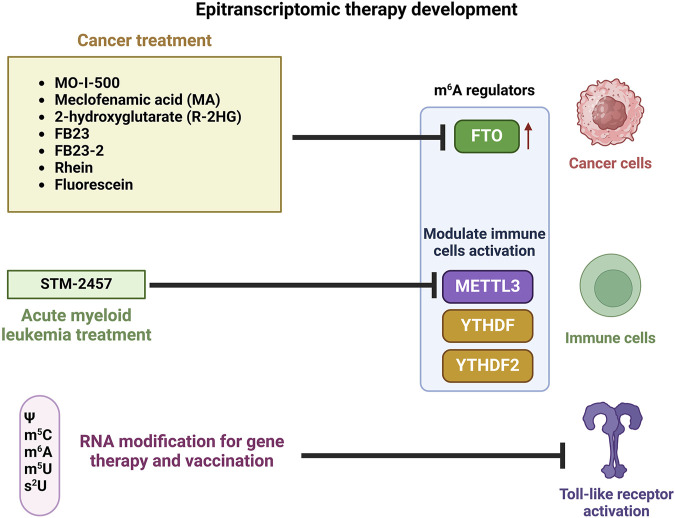
Epitranscriptomic-based therapy development for diseases. FTO (eraser), METTL3 (writer), YTHDF and YTHDF2 (readers) are m^6^A regulators. Created in BioRender. Demeny (2025) https://BioRender.com/v73i340.

To this end, it is now appreciated that numerous cellular processes are finely regulated by RNA modifications, such as RNA localization, stability, degradation, binding to other molecules, and protein biosynthesis. RNA modifications and the set of proteins involved in their installment, removal, and interpretation have been evidenced to associate with multiple types of human diseases including cancer development. Therefore, the RNA modification pathway has been considered as an ideal novel therapeutic target for treatment of various human diseases. The roles of RNA modification and related techniques in therapeutic development have been reviewed (Nombela et al., 2021; Berdasco and Esteller, 2022; Wang C. et al., 2023; Warminski et al., 2023). In this review, the current strategies and progress of epitranscriptomic-based therapeutic development are briefly highlighted (Figure 3).

As mentioned previously, m^6^A is the most prevalent internal modification in mRNA of eukaryotic species including mammals. The m^6^A modification is reversible and its dynamics are of functional importance. It has been suggested that aberrant levels of m^6^A and dysregulation of expression of its regulators (writers, erasers, and readers) are often linked to various types of cancer. Therefore, m^6^A regulators have been targeted in cancer therapies. A number of FTO inhibitors such as MO-I-500, meclofenamic acid (MA), 2-hydroxylglutarate (R-2HG), FB23, FB23-2, rhein, and fluorescein have been developed for cancer treatment (Huang et al., 2020; Li X. et al., 2022). In addition, the m^6^A regulators, METTL3, YT521-B homology domain family 1 (YTHDF1), and YTHDF2, have been indicated to modulate immune cell activation and infiltration into the tumor microenvironment and hence can influence the efficacy of immunotherapy. To develop effective strategies in targeting these regulatory proteins, a more detailed understanding on their modes of action is required (Li X. et al., 2022). A METTL3 inhibitor, STM-2457 has been reported to exhibit promising results in preclinical studies on a mouse model for AML (Berdasco and Esteller, 2022). The FDA-approved DNA methylation inhibitor, 5-azacytidine may also inhibit RNA methylation as the vast majority of 5-azacytidine is incorporated into the RNA molecule (Berdasco and Esteller, 2022). Recently, it was shown that a strategy involving inactivation of the host *YTHDF2* gene has the potential to be used to improve recombinant therapeutic protein production (Lao and Barron, 2023).

The natural form of *in vitro*-transcribed mRNAs of physiologically important proteins was considered unsuitable for clinical application because of instability. In addition, the native mRNAs activate cells of the innate immune system by stimulating TLRs. Importantly, RNA modifications through incorporation of natural nucleosides such as Ψ, m^5^C, m^6^A, 5-methyluridine (m^5^U), or 2-thiouridine (s^2^U) was demonstrated to diminish the TLRs activation (Karikó et al., 2005; Karikó et al., 2008). Moreover, in mammalian cells, mRNAs containing Ψs were found to have a higher translational capacity compared to the unmodified mRNAs, making the mRNAs harboring Ψs promising tools for gene therapy and vaccination (Karikó et al., 2008). The development of mRNA vaccines against the coronavirus disease of 2019 (COVID-19) caused by the SARS-CoV-2 was regarded as the fastest and most efficient vaccine development in human history. It should be noted that a key aspect of COVID-19 mRNA vaccines is the application of the modified nucleobase N1-methylpseudouridine (m^1^Ψ) to improve their effectiveness. Every uridine residue in the mRNA was replaced with m^1^Ψ. The m^1^Ψ nucleobase was used to enhance immune evasion and promote protein biosynthesis (Nance and Meier, 2021).

In principle, the development of mRNA therapeutics is based on the delivery of a synthetic transcript which is followed by biosynthesis of the encoded pharmacologically active protein by the cellular translational machinery (Liu and Wang, 2022). The majority of mRNA drugs is generated by *in vitro* transcription from a DNA template and can then be enzymatically modified through incorporation of modified nucleotides (Liu and Wang, 2022). Bacteriophage T7 RNA polymerase (T7 RNAP) is widely used to synthesize RNA molecules with synthetic modifications and unnatural base pairs for therapeutic purposes. It has recently been revealed that the T7 RNAP recognizes the unnatural substrates at the pre-insertion state in a different manner compared to natural substrates. This information may be useful in the generation of unnatural base pairs which are valuable for therapeutic applications (Oh et al., 2023). In addition, the use of N2 modified dinucleotide cap analogs as components of mRNA transcripts was demonstrated to enhance mRNA translation both *in vitro* and in human cells (Grzela et al., 2023). A programmable RNA base editor named RESTART has been developed for replacing uridine with Ψ in stop codons to suppress premature termination codons. This RNA-editing tool is expected to be useful in research and development of RNA-based therapeutics (Song J. et al., 2023).

## 7 Conclusion

The advancement of robust methods for detection of RNA modifications has stimulated intense research and revolutionized our understanding of multiple fundamental aspects of RNA modifications. These emerging techniques enable precise and reliable detection of the numerous modified nucleotides in RNA molecules and together with the advent of computational tools have driven the rise of the field epitranscriptomics. Improved knowledge on molecular mechanisms underlying the association of RNA modifications with various critical biological processes, including disease and its development has facilitated the construction of effective strategies for disease control and improvement of human life. Future studies should be directed towards development of more sensitive and accurate methods for detection of biochemical modifications on RNA molecules which relatively of low abundance. Detailed elucidation of molecular mechanism of each chemical modification on cellular RNA and the illumination of its biological functions remain a huge future challenge. Better understanding on distribution and signature of RNA modifications and their functional consequences is required. In addition, more accurate and reliable bioinformatics tools for data analysis need to be established. Research and development on epitranscriptomics-based therapeutics need to be strengthened in order to accelerate vaccine development and drug discovery in response to the global health issues on both communicable and noncommunicable diseases.
